# Impact of an Intervention to Promote the Vaccination of Patients with Inflammatory Bowel Disease

**DOI:** 10.3390/vaccines11111649

**Published:** 2023-10-27

**Authors:** Cristina García-Serrano, Eva Artigues-Barberà, Gloria Mirada, Pepi Estany, Joaquim Sol, Marta Ortega Bravo

**Affiliations:** 1 Atenció Primària Institut Català de la Salut, Atenció Primària, 25007 Lleida, Spain; cgarcias.lleida.ics@gencat.cat (C.G.-S.); pestany.lleida.ics@gencat.cat (P.E.); jsol.lleida.ics@gencat.cat (J.S.); 2 Multidisciplinary Research Group on Therapeutics and Interventions in Primary Care (RETICAP Group), Fundació Institut Universitari per a la Recerca a l’Atenció Primària de Salut Jordi Gol i Gurina (IDIAPJGol), Gran Via de les Corts Catalanes, 587, 08007 Barcelona, Spain; 3 Facultat d’Infermeria i Fisioteràpia, Universitat de Lleida, 25198 Lleida, Spain; gloria.mirada@gmail.com; 4 Agència de Salut Pública de Catalunya, 08005 Lleida, Spain; 5 Unitat de Suport a la Recerca de Lleida, Fundació Institut Universitari d’Investigació per a la Recerca a l’Atenció Primària de Salut Jordi Gol i Gurina (IDIAPJGol), Rambla Ferran, 44, 25007 Lleida, Spain; 6 Instituto de Investigación Biomédica de Lleida Fundación Dr. Pifarré (IRB Lleida), 25198 Lleida, Spain; 7 Facultat de Medicina, Universitat de Lleida, Pl. de Víctor Siurana, 1, 25003 Lleida, Spain

**Keywords:** inflammatory bowel disease, immunization schedule, primary health care, public health, intervention

## Abstract

Patients with inflammatory bowel disease (IBD) have a dysregulated immune system, being at high risk of opportunistic infections. Low vaccination rates hinder the prevention of such diseases. Therefore, we implemented an intervention to increase vaccination rates, and we aimed to evaluate the effect. We determined the change in professionals and the change in the vaccination rates after the intervention. A quasi-experimental study was carried out using data from 31 December 2016 to 31 December 2021. First, healthcare professionals specializing in IBD agreed on a vaccination protocol; then, this protocol was passed on to the professionals involved in vaccination. We evaluated the perception of knowledge, capacity, and intention to vaccinate patients with IBD among the professionals before and after the intervention with a survey. We also described the effectiveness of the intervention for already diagnosed patients and compared the vaccination rates between patients diagnosed prior to the intervention and newly diagnosed patients. The intervention resulted in an improved perception of knowledge, capacity, and intention to vaccinate patients with IBD among the professionals (*p* < 0.05). Moreover, during the post-intervention period, in the 315 patients, the vaccination rate increased for all immune-preventable diseases (*p* < 0.05). The professionals positively valued the intervention, and compliance with the recommended vaccination protocol in patients with IBD improved significantly.

## 1. Introduction

Immune-mediated inflammatory diseases are a group of chronic and highly disabling diseases that share common inflammatory sequences and immunological dysregulations [1]. In Spain, the prevalence of inflammatory bowel disease is 0.78%, and in Catalonia, IBD is one of the most prevalent immune-mediated inflammatory diseases, affecting almost 1% of the population [1]. The incidence of IBD in Spain is 16.2 cases per 100,000 person-years, and the incidence rate in Catalonia is 11.23 cases per 100,000 person-years [2]. This condition includes ulcerative colitis (UC), Crohn’s disease (CD), and indeterminate colitis [3,4]. IBD leads to high morbidity, mortality, and a significantly reduced quality of life [3]. Therefore, it requires medical follow-ups to reduce relapse rates and prevent complications [4].

One of the complications of immune-mediated diseases, such as IBD, is represented by opportunistic infections. These infections can take hold because of the patients’ imbalanced immune response associated with the pathology [3,5]. Infections can also occur because of the immunosuppressive treatments received [1,5,6,7,8,9,10,11,12] with the risk being more significant in the case of combined therapies [6,8,13].

Many opportunistic infections can be prevented with vaccination. However, the unhealthy immune systems of patients with IBD respond differently to vaccination [3,5,6]. Therefore, the levels of specific antigens and antibodies should be determined in each patient, adapting the vaccination protocols accordingly [3,5,6,13,14,15]. Alternatively, immunity can be built in the patients’ immediate surrounding population by vaccinating the cohabitants to reduce the transmission of infections [5,13,16]. The vaccination protocol based on the 2018 Vaccination Manual of Catalonia recommends administering the following vaccines for patients with IBD: rubella, mumps, and measles (MMR); chickenpox; tetanus; influenza; pneumococcal disease; and hepatitis B [16]. Upon the diagnosis of IBD, the patients’ vaccination history and immune status should be revised [11]. Patients who are not immunized against the above-mentioned pathogens should be advised to be vaccinated. This should occur before initiating any immunosuppressive therapy because of the risk of developing an infection consequent to such therapy [4,5,6,12,13,14,16,17]. On the other hand, the MMR and varicella vaccines are contraindicated during immunosuppressive treatments [16].

Despite the importance of vaccination as a preventive measure for infections, the vaccination rates are low in the USA, Australia, and Spain [5,10,14,18,19]. In a study conducted in the region of Lleida in 2016, the rates were lower than 64.2% in patients with IBD; therefore, vaccination is not being used to its full potential to prevent infectious diseases [20]. Up to half of the individuals with IBD may not have been aware of the need for certain vaccines [14]. Therefore, it is a priority to address patient-perceived barriers [14,17,21] and to improve the immunization rates by educating individuals and training professionals [3,11,14,17,21,22,23]. In this context, we decided to carry out an intervention for healthcare professionals involved in diagnosing and following up on patients with IBD in the region of Lleida.

The objectives of this study were as follows: (1) To evaluate the effect of our informative intervention on healthcare professionals’ perception of knowledge, capacity, and intention to vaccinate patients with IBD; (2) To compare the vaccination and immunization of patients pre- and post-intervention.

## 2. Materials and Methods

### 2.1. Design of the Study

We designed a quasi-experiment after the implementation of an informative intervention and assessed (1) its effect on healthcare professionals’ perception of knowledge, capacity, and intention to vaccinate patients with IBD and (2) its effect on the patients by comparing the vaccination rates before and after the intervention.

The intervention was aimed at managers and caregivers in gastroenterology from hospitals and PC centers in the region of Lleida who were involved in vaccination. It consisted of two phases and is further explained in Appendix A. Briefly, the phases were the following:

- Phase 1: The creation of an adapted immunization protocol by healthcare professionals involved in diagnosing and following up on patients with IBD in hospitals and PC centers. Also, a virtual consultation on vaccines was created to establish a support mechanism for the professionals who needed help or guidance regarding immunization and vaccination.
- Phase 2: Informative sessions for the immunization protocol given by experts in vaccination.
(1) Evaluation of the effect on the professionals

*Setting and study population*

The intervention was carried out between 1 October 2019 and 3 March 2020 in the region of Lleida. Informative sessions took place in hospitals and primary care (PC) centers in the same region. All professionals who attended the sessions and agreed to participate were included.

*Variables*

An evaluation survey was handed out by the research team to the professionals who attended the information sessions before and after the sessions. The research team also recorded the total number of sessions and attendees.

The survey before the sessions included the following variables:

- Self-perceived knowledge of immunization and vaccination of patients with IBD.
- Self-confidence in vaccination of patients with IBD.
- Intention to vaccinate patients with IBD.
- Opinion on the usefulness of the IBD vaccines’ virtual consultation (only after the sessions).

The results were recorded using a Likert scale (strongly disagree/disagree/undecided or indifferent/agree/strongly agree).

*Data analysis*

Welch’s paired *t*-test was used to calculate the difference between the variables before and after the intervention after assessing the non-normality and non-homogeneity of the data. The statistical significance was set at a value of *p* < 0.05.

(2) Evaluation of the effect on the patients

*Setting*

A computerized clinical history database (ECAP) from the Catalan Institute of Health was used to calculate the efficiency of the intervention in improving vaccination rates. The available data on 31 December 2021 were collected from eligible patients assigned to those PC centers in the region of Lleida that received the intervention. The prevalence of immunization in the pre-intervention period was assessed at on 31 December 2016, and it was again assessed on 31 December 2021 for the post-intervention period.

*Study population*

The study population consisted of adults with IBD in the region of Lleida. To be eligible for the study, patients had to have a clinical diagnosis of UC or CD, be 18 years of age or older, and be assigned to a PC center in the region of Lleida. Seronegative patients who had previously refused vaccination were excluded as were patients with a history of allergic reactions to a vaccine or any vaccine component necessary to achieve adequate immunization. Two groups of patients were included:

(1) Patients diagnosed before 31 December 2016. To evaluate if the professionals acted on the patients, the vaccination rates were assessed before and after the intervention in the same group of individuals.
(2) Patients diagnosed after 31 May 2017 (once the intervention started). As the vaccination of patients with IBD should be performed upon diagnosis, in this case, the vaccination rates before and after the intervention were compared using newly diagnosed patients.

*Variables*

The dependent variable was adherence to the recommended vaccination protocol. It was assessed according to compliance with the vaccination recommendations for the disease in question (UC or CD), immunosuppression status, and serological results [24,25] (Table 1). The above information was entered into a specially designed algorithm that was applied to each patient to determine whether or not there was adherence to the recommended vaccination protocol.

The independent variables were sex, age, diagnosis (UC or CD), and primary vaccination against measles, chickenpox, tetanus, influenza, pneumococcal disease, and hepatitis B [24,25].

### 2.2. Data Analysis

Quantitative variables were expressed as the mean and standard deviation (SD), and qualitative variables were expressed as absolute frequencies and percentages. The compliance with the vaccination protocol before and after the intervention was documented for all patients and classified by pathology. First, the pre- and post-intervention adherence was assessed in a cohort of patients with a diagnosis date before the intervention. We measured the effectiveness of the intervention by calculating the number of patients who moved from a poor pre-intervention adherence to a good post-intervention adherence in those vaccines in which adherence could only improve (measles, chickenpox, tetanus, and hepatitis B). The results were expressed as a percentage of patients who improved with a 95% confidence interval (CI). For those vaccines that adherence could also worsen (influenza and pneumococcal disease), a McNemar’s test was used, and the results were expressed as odds ratios (OR) and their respective 95% CI. Second, adherence was assessed in a cohort of patients with a post-intervention diagnosis date. Their adherence rates were compared to the adherence of the patients in the first cohort before the intervention. Chi-square tests were used, and odds ratios were expressed with 95% CI. The statistical significance was established at *p* < 0.05.

### 2.3. Ethics

The Ethics and Clinical Research Committee of the Fundació Institut Universitari d’Investigació per a la recerca a l’Atenció Primària de Salut Jordi Gol I Gurina (IDIAPJGol) approved this study with the code P19/212-P. The variables collected have been treated anonymously, and the confidentiality of the data is guaranteed under Regulation (EU) 2016/679 of the European Parliament and the Council on Data Protection and applicable national regulations (27 April 2016).

## 3. Results

### 3.1. Evaluation of Effect on the Professionals

A total of 134 healthcare professionals of PC attended at least one of the eight information sessions held by the vaccination expert of PC. Of these professionals, 46 were doctors (34.3%), and 88 were nurses (65.7%).

After the sessions, there was an increase of 1.48 points on the Likert scale in the perceived knowledge, 1.06 in the ability to vaccinate, and 0.34 in the intention to vaccinate (*p* < 0.05) (Table 2). Finally, the usefulness of the virtual consultation was scored with a mean of 4.62 (Table 2).

### 3.2. Evaluation of Effect on the Patients

Of the 315 patients already diagnosed with IBD included in this study, 214 (67.9%) had been diagnosed with UC, and 101 (32.1%) had been diagnosed with CD. Their mean (SD) age was 49.4 (15.6) years, and 153 (48.6%) were female (Table 3).

**Figure 1:**
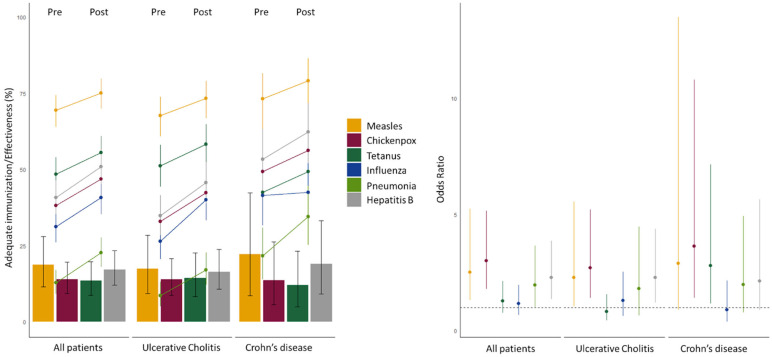
Effect of the intervention on the patients. **Left panel**: Effect on the patients diagnosed before intervention. Bars represent the effectiveness of the intervention, expressed as the % of patients that improved their immunization status. Dots represent the % of patients that had an adequate immunization before and after the intervention. Lines represent the 95% confidence intervals. **Right panel**: Effect on the newly diagnosed patients. Dots express the odds ratios of newly diagnosed patients having an adequate immunization compared with those patients diagnosed before the intervention. Lines represent the 95% confidence intervals.

An increase in the vaccination rate of these patients was observed after the intervention for all immune-preventable diseases, ranging from 2.58% to 18.80% (Table 4, Figure 1). The implementation of an IBD protocol helped improve the vaccination rates for patients who were already diagnosed at the beginning of the study. This means that the health professionals reviewed the vaccination status of the patients they had already identified.

Also, there was an increase in compliance with the vaccination protocol when comparing patients diagnosed before the intervention to those diagnosed afterwards. Considering the whole population, the increase was significant in the vaccinations for measles, chickenpox, and hepatitis B (Table 5, Figure 1). The effect of the intervention was greater in the vaccines recommended by risk group and not by age, which could be due to the greater awareness of the professionals with the implementation of a specific protocol in our healthcare region. In the case of influenza, the effect was not observed, which could probably be related to the type of routine recruitment by vaccination campaign that is mostly carried out by age.

Another important factor was the increase in the administration of live attenuated vaccines at the time of diagnosis to patients. The new diagnoses in 2022 showed an increase in vaccination coverage against measles and chickenpox. This is important because said vaccination could be compromised once immunosuppressive treatment for IBD is started.

Stratified results by sex and age group can be found in the supplementary materials (Supplementary Tables S1–S4). In relation to the effect of the intervention based on sex, greater changes are observed in women regardless of the time of their diagnosis before or after the intervention (Supplementary Tables S1 and S2). However, in CD, greater effectiveness is observed in men, which could be due to the fact that they have much lower vaccine coverage than women.

Moreover, we observed that, in age stratification, there is a greater effect in patients already diagnosed with IBD at older ages (Supplementary Table S3). This could be due to the greater awareness of health professionals in vaccination by age. On the other hand, in recently diagnosed patients, a greater effect is observed at younger ages, so the intervention has had a positive effect in terms of vaccination by risk group and not by age (Supplementary Table S4). Despite this, there has been improvement in all age groups in both cases.

Another relevant datum is the greater effect of the intervention in CD in patients less than or equal to 40 years of age already diagnosed and in patients aged between 41 and 60 years with a recent diagnosis, which could be due to the greater severity of the disease that leads to the use of immunosuppressant treatment.

## 4. Discussion

### 4.1. Main Findings

Opportunistic infections are one of the complications of IBD. Although vaccination is a primary prevention measure for these infections, the vaccination rates in clinical practice are low [5,10,14,18]. A recent study in Chile showed that 23% of the patients with IBD who were included had no vaccination against any diseases [26]. In the region of Lleida, less than 65% of 1722 patients with UC or CD complied with the vaccination guidelines [20].

The main problems causing the low vaccination rates in patients with IBD are a lack of knowledge of the vaccination protocol, contraindications, and side effects by the professionals; a mistrust of vaccines; and poor communication between the professionals [10,14,21,26]. Bianchi et al. [27] carried out a systematic review of the COVID-19 immunization rates in IBD in which they described, as negative determinants towards vaccination, the fear of exacerbation of the disease after administering the COVID-19 vaccine or its adverse effects and the lack of information. Even patients on immunosuppressive treatment preferred to avoid vaccination. Some positive determinants were a self-perceived higher risk for COVID-19 in patients with IBD, herd protection, and having a history of receiving prior vaccinations, particularly the influenza vaccine. Furthermore, with respect to gender, higher levels of compliance were observed in women, as occurred in our study with other vaccines.

Also, patients are unaware of the need for certain vaccines [14], which could be related to the information imparted by the professionals [28], for which reason it was contemplated in the protocol to inform them about the recommended vaccines and their accessibility. Indeed, Wasan et al. surveyed gastroenterologists and found that approximately half of the professionals did not ask their patients about their vaccination history, delegating the prescription and administration of vaccines to PC centers [10]. Furthermore, it was observed that 30% of the professionals did not follow the recommended guidelines for immunocompromised patients [10]. Therefore, having a virtual consultation with vaccination experts to increase safety in clinical practice is justified. In our study, this tool was considered very useful by the professionals working in PC centers to resolve doubts regarding the immunization and vaccination of people with IBD. Several authors state that the vaccination levels are suboptimal in hospitals and PC centers [5,10,11,14,15]. In particular, in the region of Lleida, most patients are treated in hospitals; however, vaccinations are carried out in PC centers and recorded in the medical record database. Many patients do not get vaccinated in hospitals and do not go to PC centers for vaccination [14,21]. Furthermore, in PC centers, there are no clear guidelines for vaccination. This directly contributes to the low vaccination rates and the consequent risk of opportunistic infections [5,14,20].

Murdaca et al. defend that the promotion of prevention strategies such as vaccination is ethical and cost effective, and every physician should actively carry it out in immunomediated diseases such as systemic sclerosis [28]. The authors reported that the available literature showed a lower incidence of opportunistic infections and complications in immunocompromised patients and demonstrated the safety of the vaccines in these patients, reinforcing the importance of interventions focused on improving their vaccination coverage [28]. A multidisciplinary protocol was implemented in hospitals and PC centers in the Lleida region to clarify the best moment to vaccinate the patients and the vaccination workflow. To achieve health maintenance goals in IBD, it is necessary to coordinate between PC centers, gastroenterologists, public health experts, and other specialists [27,29]. Furthermore, Yamamoto et al. indicated the need to strengthen the role of PC centers in the vaccination and follow-up of patients with IBD [21]. Therefore, addressing the barriers perceived by professionals and patients is already a priority, as observed in the implementation of professional information programs [10,30]. However, few interventions included the PC setting [31]. A systematic review of the interventions used to improve adherence to preventive care in IBD concluded that targeting gastroenterologists, patients, or both was an effective strategy [31,32,33,34].

Bellali et al. concluded that treating factors, such as intention and motivation, in the health professional can be used to increase vaccine coverage [35]. We describe the positive effect of our intervention on healthcare professionals to improve their perceived knowledge, ability, and intention to vaccinate patients with IBD as well as to increase the vaccination rates of these patients in the region of Lleida. In addition, when looking at patients diagnosed with IBD following the intervention, we saw an improvement in the immunization and vaccination rates in comparison to those diagnosed before the intervention. We also found that having a virtual consultation with vaccination experts was considered useful by the PC professionals to resolve doubts regarding the immunization and vaccination of people with IBD.

### 4.2. Strengths and Limitations

The main strength of our study is that our data include all the diagnosed patients in the healthcare region, which grants the representativity of the results. However, our data may be subject to reporting bias because of incomplete records regarding vaccination and immunization criteria, as they are based on a database (ECAP) from the Catalan Institute of Health. This bias should not have changed between the pre- and post-intervention analysis and, therefore, should not affect this study.

Our intervention was also limited by the COVID-19 pandemic since, at that time, information sessions were being held for the professionals in PC centers. Patients from the PC centers where the intervention was not performed were excluded to reduce information bias.

### 4.3. Implications

Impaired immunogenicity in patients with IBD and low vaccination rates are major public health problems. It is crucial to identify and address the barriers to vaccination. Our results confirm that informative interventions aimed at professionals can improve compliance with the recommended vaccination protocol.

## 5. Conclusions

The results of this study clarify crucial aspects of healthcare delivery and the impact of interventions on the vaccination practices of patients with IBD. One of the most relevant results of the intervention was the significant improvement in compliance with the recommended vaccination protocol among patients with IBD. Furthermore, the healthcare professionals positively valued their perception of knowledge, ability, and intention to vaccinate after the intervention led by nurses with experience in vaccination. A notable element of the intervention was the introduction of virtual vaccine consultations led by nurses with expertise in vaccinations. This approach was favorably received by the healthcare professionals, highlighting the potential benefits of incorporating these types of virtual modalities into healthcare services, which provide the immediate solutions required in IBD due to the short optimal vaccination period and would contribute to the aim of a high vaccination coverage from a public health point of view. We can also affirm that, with the right strategies, positive changes in vaccination practices can be achieved. However, it should be noted that there is still room for improvement in the vaccination rates and immunization coverage of these patients; so, there are still additional barriers or challenges that need to be addressed.

## Figures and Tables

**Table 1:** Criteria for assessing compliance to vaccination.

Infections	Event Identification (At Least One of the Following Criteria Must Be Met)
Measles	Date of birth prior to 1 January 1966. A history of measles. A seropositivity level for the measles virus >16.5 IU/mL. 2 doses of a vaccine containing the measles antigen having been administered at least 1 month apart.
Chickenpox	A history of chickenpox. A seropositivity level for the varicella-zoster virus IgG > 165 IU/mL. 2 doses of the varicella vaccine having been administered at least 1 month apart.
Tetanus	At least 5 doses of the tetanus toxoid vaccine having been administered before age 16 years. At least 3 doses of the tetanus toxoid vaccine having been administered with a minimum interval of 0, 1, and 6 months after age 7 years.
Influenza	Administration of the influenza vaccine (a high-dose seasonal influenza vaccine for patients over 60 or 65 years of age or a standard influenza vaccine for other patients).
Pneumococcal disease	1 dose of pneumococcal 13-valent conjugate vaccine (PCV13) and 1 dose of pneumococcal polysaccharide vaccine (PPSV23) having been administered at least 2 months apart. At least 1 dose of PPSV23 and one dose of PCV13 having been administered at least 12 months apart. At least 1 dose of PCV13 or PPSV23 having been administered in the last year.
Hepatitis B	A history of hepatitis B. A seropositivity level for the hepatitis B surface antigen (HBsAg) >0.9 IU/mL; total antibody (anti-HBc) > 0.9 IU/mL. A post-vaccination seropositivity level for anti-HBs > 12 IU/mL ^a^. At least 3 doses of the hepatitis B vaccine having been administered with a minimal interval of 0, 1, and 6 months. At least 4 doses of the hepatitis B vaccine having been administered with a minimum interval of 0, 1, 2, and 6 months. At least 3 doses of the hepatitis A + B vaccine having been administered with a minimum interval of 0, 1, and 6 months.

Immunoglobulin G = IgG. ^a^ According to the laboratory criteria of the Lleida region, Spain.

**Table 2:** Comparison before–after the intervention for the professionals.

	Pre-Intervention	Post-Intervention	Difference (Post–Pre)	Paired *t*-Test (Post vs. Pre)	*N*
Perception of knowledge	2.90 (0.98)	4.38 (0.70)	1.48 (1.07)	<0.001	133
Ability to vaccinate	2.95 (1.06)	4.01 (0.79)	1.06 (0.96)	<0.001	132
Intention to vaccinate	4.03 (1.06)	4.39 (0.75)	0.36 (0.90)	<0.001	131
Perceived usefulness of the virtual consultation		4.62 (0.64)			130

All values are expressed as means and standard deviations.

**Table 3:** Socio-demographic characteristics of the patients already diagnosed with IBD included in this study.

	[Total]
	*N* = 315
Sex:	
Female	152 (48.3%)
Male	163 (51.7%)
Age, mean (SD)	49.4 (15.6)
Age group	
≤40	99 (31.4%)
41–60	68 (21.6%)
>60	148 (47.0%)
Pathology:	
UC	214 (37.9%)
CD	101 (32.1%)

**Table 4:** A comparison of the adherence to the recommended vaccination protocol before and after the intervention in patients already diagnosed with IBD.

	Total				UC				CD			
	2017	2022	Effectiveness	*p*	2017	2022	Effectiveness	*p*	2017	2022	Effectiveness	*p*
	*N* = 315	*N* = 315			*N* = 214	*N* = 214			*N* = 101	*N* = 101		
Measles	219 (69.5%)	237 (75.2%)	18.8% [11.5%; 28.0%]	-	145 (67.8%)	157 (73.4%)	17.4% [9.32%; 28.4%]	-	74 (73.3%)	80 (79.2%)	22.2% [8.62%; 42.3%]	-
Chickenpox	121 (38.4%)	148 (47.0%)	13.9% [9.38%; 19.6%]	-	71 (33.2%)	91 (42.5%)	14.0% [8.76%; 20.8%]	-	50 (49.5%)	57 (56.4%)	13.7% [5.70%; 26.3%]	-
Tetanus	153 (48.6%)	175 (55.6%)	13.6% [8.71%; 19.8%]	-	110 (51.4%)	125 (58.4%)	14.4% [8.30%; 22.7%]	-	43 (42.6%)	50 (49.5%)	12.1% [4.99%; 23.3%]	-
Influenza	99 (31.4%)	129 (41.0%)	2.58 [1.49; 4.64]	<0.001	57 (26.6%)	86 (40.2%)	5.14 [2.26; 13.69]	<0.001	42 (41.6%)	43 (42.6%)	1.08 [0.46; 2.6]	1
Pneumococcal disease	41 (13.0%)	72 (22.9%)	4.44 [2.12; 10.42]	<0.001	19 (8.88%)	37 (17.3%)	4 [1.59; 11.96]	0.001	22 (21.8%)	35 (34.7%)	5.33 [1.53; 28.56]	0.004
Hepatitis B	129 (41.0%)	161 (51.1%)	17.2% [12.1%; 23.4%]	-	75 (35.0%)	98 (45.8%)	16.5% [10.8%; 23.8%]	-	54 (53.5%)	63 (62.4%)	19.1% [9.15%; 33.3%]	-

**Table 5:** A comparison of the adherence to the recommended vaccination protocol between patients diagnosed with IBD before and after the intervention.

	Total					UC					CD				
	2017	2022	Effectiveness	*p* Ratio	*p* Overall	2017	2022	Effectiveness	*p* Ratio	*p* Overall	2017	2022	Effectiveness	*p* Ratio	*p* Overall
	*N* = 315	*N* = 75				*N* = 214	*N* = 47				*N* = 101	*N* = 28			
Measles	219 (69.5%)	64 (85.3%)	2.5 [1.3; 5.3]	0.004	0.009	145 (67.8%)	39 (83.0%)	2.3 [1.1; 5.6]	0.035	0.058	74 (73.3%)	25 (89.3%)	2.9 [0.9; 13.5]	0.074	0.128
Chickenpox	121 (38.4%)	49 (65.3%)	3.0 [1.8; 5.2]	<0.001	<0.001	71 (33.2%)	27 (57.4%)	2.7 [1.4; 5.2]	0.002	0.003	50 (49.5%)	22 (78.6%)	3.6 [1.4; 0.8]	0.006	0.012
Tetanus	153 (48.6%)	41 (54.7%)	1.3 [0.8; 2.1]	0.347	0.412	110 (51.4%)	22 (46.8%)	0.8 [0.4; 1.56]	0.574	0.682	43 (42.6%)	19 (67.9%)	2.8 [1.2; 7.2]	0.02	0.031
Influenza	99 (31.4%)	26 (34.7%)	1.2 [0.7;2.0]	0.588	0.687	57 (26.6%)	15 (31.9%)	1.3 [0.6; 2.5]	0.466	0.58	42 (41.6%)	11 (39.3%)	0.9 [0.4; 2.2]	0.836	0.999
Pneumococcal disease	41 (13.0%)	17 (22.7%)	2.0 [1.0; 3.7]	0.044	0.054	19 (8.9%)	7 (14.9%)	1.8 [0.7; 4.47]	0.233	0.278	22 (21.8%)	10 (35.7%)	2.0 [0.8; 4.9]	0.148	0.207
Hepatitis B	129 (41.0%)	46 (61.3%)	2.3 [1.4; 3.9]	0.002	0.002	75 (35.0%)	26 (55.3%)	2.3 [1.2; 4.4]	0.012	0.016	54 (53.5%)	20 (71.4%)	2.1 [0.9; 5.7]	0.093	0.138

## Data Availability

Data available by request.

## Supplementary Materials

The following supporting information can be downloaded at: https://www.mdpi.com/article/10.3390/vaccines11111649/s1, Table S1: A comparison of the adherence to the recommended vaccination protocol before and after the intervention in patients already diagnosed with IBD stratified by sex; Table S2: A comparison of the adherence to the recommended vaccination protocol between patients diagnosed with IBD before and after the intervention stratified by sex; Table S3: A comparison of the adherence to the recommended vaccination protocol before and after the intervention in patients already diagnosed with IBD stratified by age group; Table S4: A comparison of the adherence to the recommended vaccination protocol between patients diagnosed with IBD before and after the intervention stratified by age group.

## Appendix A. Intervention

Phase 1: Consensus and support tools among healthcare professionals involved in diagnosing and following up on patients with IBD in hospitals and primary care (PC) centers in the region of Lleida.

The first phase involved raising awareness of the importance of immunization and vaccination. Subsequently, several meetings were held between representatives of the management, heads of the care management units, and digestive services of the two referral hospitals in the region of Lleida. A multidisciplinary protocol adapted to hospitals and PC was created and internally accepted. This protocol was entitled “Protocol d’immunització en pacients candidats a therapies biològiques i/o immunosupressors” (Immunization protocol in patient candidates for biological and/or immunosuppressive therapies). This information was circulated widely. Also, a virtual vaccine consultation was created to establish a support mechanism for the professionals who needed to resolve doubts regarding immunization and vaccination. Through the computerized clinical history (ECAP), the healthcare professionals had access to the patient’s vaccination schedule, and if not already in place, they could establish an individualized one.

Phase 2: Briefing of the primary care professionals involved in the vaccination process.

In Phase 2, the information from Phase 1 was communicated to the professionals involved in the vaccination process. A two-hour information session was conducted by vaccination experts of PC in each hospital and PC center of the region of Lleida. The session was intended to educate staff about the vaccination protocol to improve immunization and vaccination rates in patients with IBD and the current guidelines recommended in the 2018 Vaccination Manual of Catalonia. The virtual vaccine consultation tool was also explained.

Before and after the session, a Likert-scale survey (strongly disagree/disagree/undecided/ agree/strongly agree) was carried out to assess the self-perceived knowledge and the ability and intention of professionals to vaccinate patients. After the session, the usefulness of the virtual vaccine consultation for professionals was assessed.

## References

[1] Puig L., Ruiz de Morales J.G., Dauden E., Andreu J.L., Cervera R., Adán A., Marsal S., Escobar C., Hinojosa J., Palau J. (2019). La prevalencia de diez enfermedades inflamatorias inmunomediadas (IMID) en España [Prevalence of ten immune-mediatedinflammatorydiseases (IMIDs) in Spain]. Rev. Esp. Salud Pública.

[2] Chaparro M., Garre A., Núñez Ortiz A., Diz-Lois Palomares M.T., Rodríguez C., Riestra S., Vela M., Benítez J.M., Fernández Salgado E., Sánchez Rodríguez E. (2021). Incidence, Clinical Characteristics and Management of Inflammatory Bowel Disease in Spain: Large-Scale Epidemiological Study. J. Clin. Med..

[3] Oltra L., Casellas F. (2016). Enfermedad Inflamatoria Intestinal para Enfermería [Inflammatory Bowel Disease for Nursing].

[4] Riestra Menéndez S., de Francisco García R., Pérez-Martínez I. (2012). Manejo extra-hospitalario de la enfermedad inflamatoria intestinal: Papel de Atención Primaria. Med. Programa Form. Méd. Contin. Acreditado.

[5] Campins M., Cossio Y., Martínez X., Borruel N. (2013). Vaccination of patients with inflammatory bowel disease: Practical recommendations. Rev. Española Enfermedades Dig..

[6] Rahier J.F., Magro F., Abreu C., Armuzzi A., Ben-Horin S., Chowers Y., Cottone M., de Ridder L., Doherty G., Ehehalt R. (2014). Second European evidence-based consensus on the prevention, diagnosis and management of opportunistic infections in inflammatory bowel disease. J. Crohn’s Colitis.

[7] Bernal I., Domènech E., García-Planella E., Cabré E., Gassull M.A. (2003). Infecciones oportunistas en pacientes con enfermedad inflamatoria intestinal bajo tratamiento inmunosupresor. Gastroenterol. Hepatol..

[8] Toruner M., Loftus E.V., Harmsen W.S., Zinsmeister A.R., Orenstein R., Sandborn W.J., Colombel J.F., Egan L.J. (2008). Risk Factors for Opportunistic Infections in Patients with Inflammatory Bowel Disease. Gastroenterology.

[9] Rahier J.F., Ben-Horin S., Chowers Y., Conlon C., De Munter P., D’Haens G., Domènech E., Eliakim R., Eser A., Frater J. (2009). European evidence-based Consensus on the prevention, diagnosis and management of opportunistic infections in inflammatory bowel disease. J. Crohn’s Colitis.

[10] Wasan S.K., Coukos J.A., Farraye F.A. (2011). Vaccinating the inflammatory bowel disease patient: Deficiencies in gastroenterologists’ knowledge. Inflamm. Bowel Dis..

[11] Viget N., Vernier-Massouille G., Salmon-Ceron D., Yazdanpanah Y., Colombel J.F. (2008). Opportunistic infections in patients with inflammatory bowel disease: Prevention and diagnosis. Gut.

[12] Mazzola G., Macaluso F.S., Adamoli L., Renna S., Cascio A., Orlando A. (2017). Diagnostic and vaccine strategies to prevent infections in patients with inflammatory bowel disease. J. Infect..

[13] Bühler S., Hatz C. Background Document on Immune-Mediated Inflammatory Diseases (IMID) Module 2 Vaccination in Patients with Autoimmune Inflammatory Rheumatic Diseases (AIIRD).

[14] Gurvits G.E. (2018). Lax Prophylaxis: Vaccinating the Inflammatory Bowel Disease Patient. Dig. Dis. Sci..

[15] Kochar B., Herfarth H.H. (2018). Vaccinations in Adult Patients with Inflammatory Bowel Diseases in the West. Inflamm. Intest. Dis..

[16] Agència de Salut Pública de la Generalitat de Catalunya (2018). Manual de Vacunacions de Catalunya [Vaccination Manual of Catalonia].

[17] Wasan S., Baker S., SkolniK P., Farray F.A. (2010). A practical guide to vaccinating the inflammatory bowel disease patient. Am. J. Gastroenterol..

[18] Marín A.C., Gisbert J.P., Chaparro M. (2015). Immunogenicity and mechanisms impairing the response to vaccines in inflammatory bowel disease. World J. Gastroenterol..

[19] Crawford N.W., Catto-Smith A.G., Oliver M.R., Cameron D.J., Buttery J.P. (2011). An Australian audit of vaccination status in children and adolescents with inflammatory bowel disease. BMC Gastroenterol..

[20] García-Serrano C., Mirada G., Marsal J.R., Ortega M., Sol J., Solano R., Artigues E.M., Estany P. (2020). Compliance with the guidelines on recommended immunization schedule in patients with inflammatory bowel disease: Implications on public health policies. BMC Public Health.

[21] Yamamoto-Furusho J.K., Sarmiento-Aguilar A., Parra-Holguín N.N., Bozada-Gutiérrez K.E. (2018). Evaluación del esquema de vacunación y cuidados con relación al seguimiento y tratamiento de los pacientes con enfermedad inflamatoria intestinal [Assessment of thevaccination and careschedule in relation to thefollow-up and treatment of patientswithinflammatoryboweldisease]. Rev. Gastroenterol. Mex..

[22] Martin S., Bryant R. (2019). Opportunistic Infections. Inflammatory Bowel Disease Nursing Manual.

[23] Coenen S., Weyts E., Jorissen C., De Munter P., Noman M., Ballet V., Vermeire S., Van Assche G., Ferrante M. (2017). Effects of Education and Information on Vaccination Behavior in Patients with Inflammatory Bowel Disease. Inflamm. Bowel Dis..

[24] Departament de Salut (2016). Calendari de Vacunacions sistemàtiques [Department of Health. Generalitat de Catalunya. Systematic Vaccination Schedule].

[25] Campins M., Martínez X., Cossio Y. (2012). Protocolos de la sempsph autores: Fecha de revisión: Protocolo de vacunación de pacientes con enfermedad inflamatoria intestinal [protocolforvaccination of patientswithinflammatoryboweldisease]. https://www.sempspgs.es/index.php?menu=68&idioma=es&buscarseccion=2012.

[26] Quera R., Simian D., Núñez P., Flores L., Figueroa C., Ibáñez P., Kronberg U., Lubascher J., Pizarro G. (2021). ¿Están recibiendo los pacientes con enfermedad inflamatoria intestinal una adecuada inmunización? [Are patients with inflammatory bowel disease receiving adequate immunisation?] Gastroenterol. Hepatol..

[27] Bianchi F.P., Donghia R., Tatoli R., Bonfiglio C. (2023). COVID-19 Immunization Rates in Patients with Inflammatory Bowel Disease Worldwide: A Systematic Review and Meta-Analysis. Vaccines.

[28] Murdaca G., Noberasco G., Olobardi D., Lunardi C., Maule M., Delfino L., Triggiani M., Cardamone C., Benfaremo D., Moroncini G. (2021). Current Take on Systemic Sclerosis Patients’ Vaccination Recommendations. Vaccines.

[29] Farraye F.A., Melmed G.Y., Lichtenstein G.R., Kane V.K. (2017). ACG Clinical Guideline: Preventive Care in Inflammatory Bowel Disease. Am. J. Gastroenterol..

[30] Christensen K.R., Steenholdt C., Buhl S.S., Ainsworth M.A., Thomsen O., Brynskov J. (2015). Systematic Information to Health-Care Professionals about Vaccination Guidelines Improves Adherence in Patients with Inflammatory Bowel Disease in Anti-TNFα Therapy. Am. J. Gastroenterol..

[31] Huth K., Benchimol E.I., Aglipay M., Mack D.R. (2015). Strategies to Improve Influenza Vaccination in Pediatric Inflammatory Bowel Disease Through Education and Access. Inflamm. Bowel Dis..

[32] Parker S., White L.C., Spangler C., Rosenblum J., Sweeney S., Homan E., Bensen S.P., Levy L.C., Dragnev M.C., Moskalenko-Locke K. (2013). A quality improvement project significantly increased the vaccination rate for immunosuppressed patients with IBD. Inflamm. Bowel Dis..

[33] Yu N., Basnayake C., Connell W., Ding N.S., Wright E., Stanley A., Fry S., Wilson-O’Brien A., Niewiadomski O., Lust M. (2022). Interventions to Improve Adherence to Preventive Care in Inflammatory Bowel Disease: A Systematic Review. Inflamm. Bowel Dis..

[34] Abreu C., Martins A., Silva F., Canelas G., Ribeiro L., Pinto S., Sarmento A., Magro F. (2023). Adherence to Vaccines in Adult Patients with Immune-Mediated Inflammatory Diseases: A Two-Year Prospective Portuguese Cohort Study. Vaccines.

[35] Bellali T., Liamopoulou P., Karavasileiadou S., Almadani N., Galanis P., Kritsotakis G., Manomenidis G. (2023). Intention, Motivation, and Empowerment: Factors Associated with Seasonal Influenza Vaccination among Healthcare Workers (HCWs). Vaccines.

