# Supplementary material for: Impact of an Intervention to Promote the Vaccination of Patients with Inflammatory Bowel Disease
Source: Vaccines (Basel). 2023 Oct 27;11(11):1649. doi: 10.3390/vaccines11111649 (PMC10674651; doi:10.3390/vaccines11111649)
Supplement: Supplementary file 1 [file vaccines-11-01649-s001.zip › vaccines-2663975-supplementary.pdf]

Supplementary Table S1. A comparison of the adherence to the recommended vaccination protocol before and after the intervention in patients already diagnosed with IBD stratified by sex.

	Total				UC				CD			
					Women							
	2017	2022	Effectiveness	p-value	2017	2022	Effectiveness	p-value	2017	2022	Effectiveness	p-value
	N=152	*N=152*			*N=110*	*N=110*			*N=42*	*N=42*		
Measles	110 (72.4%)	119 (78.3%)	21.4% [10.3%;36.8%]	-	77 (70.0%)	85 (77.3%)	24.2% [11.1%;42.3%]	-	33 (78.6%)	34 (81.0%)	11.1% [0.28%;48.2%]	-
Chickenpox	65 (42.8%)	78 (51.3%)	14.9% [8.20%;24.2%]	-	41 (37.3%)	53 (48.2%)	17.4% [9.32%;28.4%]	-	24 (57.1%)	25 (59.5%)	5.56% [0.14%;27.3%]	-
Tetanus	80 (52.6%)	94 (61.8%)	19.4% [11.1%;30.5%]	-	59 (53.6%)	69 (62.7%)	19.6% [9.82%;33.1%]	-	21 (50.0%)	25 (59.5%)	19.0% [5.45%;41.9%]	-
Influenza	51 (33.6%)	76 (50.0%)	4.13 [1.87;10.34]	<0.001	31 (28.2%)	53 (48.2%)	6.5 [2.26;25.63]	<0.001	20 (47.6%)	23 (54.8%)	1.75 [0.44;8.15]	0,549
Pneumococcal disease	21 (13.8%)	38 (25.0%)	5.25 [1.77;21.04]	0,001	12 (10.9%)	23 (20.9%)	4.67 [1.3;25.33]	0,013	9 (21.4%)	15 (35.7%)	7 [0.9;315.48]	0,07
Hepatitis B	62 (40.8%)	80 (52.6%)	20.0% [12.3%;29.8%]	-	39 (35.5%)	55 (50.0%)	22.5% [13.5%;34.0%]	-	23 (54.8%)	25 (59.5%)	10.5% [1.30%;33.1%]	-
					Men							
	2017	2022	Effectiveness	p-value	2017	2022	Effectiveness	p-value	2017	2022	Effectiveness	p-value
	N=163	*N=163*			*N=104*	*N=104*			*N=59*	*N=59*		
Measles	109 (66.9%)	118 (72.4%)	16.7% [7.92%;29.3%]	-	68 (65.4%)	72 (69.2%)	11.1% [3.11%;26.1%]	-	41 (69.5%)	46 (78.0%)	27.8% [9.69%;53.5%]	-

Chickenpox	56 (34.4%)	70 (42.9%)	13.1% [7.34%;21.0%]	-	30 (28.8%)	38 (36.5%)	10.8% [4.78%;20.2%]	-	26 (44.1%)	32 (54.2%)	18.2% [6.98%;35.5%]	-
Tetanus	73 (44.8%)	81 (49.7%)	8.89% [3.92%;16.8%]	-	51 (49.0%)	56 (53.8%)	9.43% [3.13%;20.7%]	-	22 (37.3%)	25 (42.4%)	8.11% [1.70%;21.9%]	-
Influenza	48 (29.4%)	53 (32.5%)	1.45 [0.63;3.47]	0,442	26 (25.0%)	33 (31.7%)	3.33 [0.86;18.85]	0,092	22 (37.3%)	20 (33.9%)	0.75 [0.21;2.46]	0,791
Pneumococcal disease	20 (12.3%)	34 (20.9%)	3.8 [1.37;13.02]	0,007	7 (6.73%)	14 (13.5%)	3.33 [0.86;18.85]	0,092	13 (22.0%)	20 (33.9%)	4.5 [0.93;42.8]	0,065
Hepatitis B	67 (41.1%)	81 (49.7%)	14.6% [8.21%;23.3%]	-	36 (34.6%)	43 (41.3%)	10.3% [4.24%;20.1%]	-	31 (52.5%)	38 (64.4%)	25.0% [10.7%;44.9%]	-

Supplementary Table S2. A comparison of the adherence to the recommended vaccination protocol between patients diagnosed with IBD before and after the intervention stratified by sex.

	**Total**					**UC**					**CD**				
	**Women**														
	**2017**	**2022**	**OR**	**p ratio**	**p overall**	**2017**	**2022**	**OR**	**p ratio**	**p overall**	**2017**	**2022**	**OR**	**p ratio**	**p overall**
	N=152	*N=40*				*N=110*	*N=28*				*N=42*	*N=12*			
Measles	110 (72.4%)	36 (90.0%)	3.32 [1.22;11.9]	0,016	0,034	77 (70.0%)	25 (89.3%)	3.41 [1.08;15.7]	0,034	0,067	33 (78.6%)	11 (91.7%)	2.66 [0.41;72.0]	0,347	0,426
Chickenpox	65 (42.8%)	28 (70.0%)	3.08 [1.48;6.77]	0,002	0,004	41 (37.3%)	18 (64.3%)	2.99 [1.27;7.39]	0,012	0,018	24 (57.1%)	10 (83.3%)	3.49 [0.77;27.2]	0,109	0,174
Tetanus	80 (52.6%)	23 (57.5%)	1.21 [0.60;2.49]	0,59	0,71	59 (53.6%)	14 (50.0%)	0.87 [0.37;2.01]	0,735	0,895	21 (50.0%)	9 (75.0%)	2.86 [0.72;15.1]	0,141	0,227
Influenza	51 (33.6%)	14 (35.0%)	1.07 [0.50;2.21]	0,857	1	31 (28.2%)	10 (35.7%)	1.42 [0.57;3.40]	0,444	0,584	20 (47.6%)	4 (33.3%)	0.56 [0.13;2.14]	0,407	0,583
Pneumococcal disease	21 (13.8%)	6 (15.0%)	1.12 [0.38;2.87]	0,828	1	12 (10.9%)	3 (10.7%)	1.01 [0.21;3.55]	0,985	1	9 (21.4%)	3 (25.0%)	1.24 [0.22;5.42]	0,784	1
Hepatitis B	62 (40.8%)	22 (55.0%)	1.77 [0.87;3.62]	0,113	0,152	39 (35.5%)	14 (50.0%)	1.81 [0.77;4.25]	0,169	0,232	23 (54.8%)	8 (66.7%)	1.61 [0.43;7.10]	0,489	0,686
	**Men**														
	**2017**	**2022**	**OR**	**p ratio**	**p overall**	**2017**	**2022**	**OR**	**p ratio**	**p overall**	**2017**	**2022**	**OR**	**p ratio**	**p overall**
	N=163	*N=35*				*N=104*	*N=19*				*N=59*	*N=16*			
Measles	109 (66.9%)	28 (80.0%)	1.95 [0.83;5.16]	0,128	0,185	68 (65.4%)	14 (73.7%)	1.45 [0.50;4.90]	0,503	0,659	41 (69.5%)	14 (87.5%)	2.87 [0.69;21.5]	0,159	0,208

Chickenpox	56 (34.4%)	21 (60.0%)	2.84 [1.35;6.16]	0,006	0,008	30 (28.8%)	9 (47.4%)	2.21 [0.79;6.10]	0,128	0,184	26 (44.1%)	12 (75.0%)	3.66 [1.11;14.8]	0,032	0,056
Tetanus	73 (44.8%)	18 (51.4%)	1.30 [0.62;2.74]	0,481	0,597	51 (49.0%)	8 (42.1%)	0.76 [0.27;2.06]	0,592	0,759	22 (37.3%)	10 (62.5%)	2.74 [0.88;9.25]	0,083	0,128
Influenza	48 (29.4%)	12 (34.3%)	1.25 [0.56;2.70]	0,572	0,717	26 (25.0%)	5 (26.3%)	1.09 [0.32;3.20]	0,884	1	22 (37.3%)	7 (43.8%)	1.31 [0.41;4.08]	0,646	0,856
Pneumococcal disease	20 (12.3%)	11 (31.4%)	3.26 [1.35;7.66]	0,01	0,01	7 (6.73%)	4 (21.1%)	3.69 [0.84;14.2]	0,08	0,067	13 (22.0%)	7 (43.8%)	2.71 [0.81;8.92]	0,103	0,112
Hepatitis B	67 (41.1%)	24 (68.6%)	3.09 [1.44;7.01]	0,003	0,006	36 (34.6%)	12 (63.2%)	3.18 [1.16;9.37]	0,024	0,037	31 (52.5%)	12 (75.0%)	2.62 [0.79;10.6]	0,117	0,185

Supplementary Table S3. A comparison of the adherence to the recommended vaccination protocol before and after the intervention in patients already diagnosed with IBD stratified by age group.

	**Total**				**UC**				**CD**			
						**≤40 years old**						
	**2017**	**2022**	**Effectiveness**	**p-value**	**2017**	**2022**	**Effectiveness**	**p-value**	**2017**	**2022**	**Effectiveness**	**p-value**
	N=99	*N=99*			*N=54*	*N=54*			*N=45*	*N=45*		
Measles	57 (57.6%)	63 (63.6%)	14.3% [5.43%;28.5%]	-	27 (50.0%)	28 (51.9%)	3.70% [0.09%;19.0%]	-	30 (66.7%)	35 (77.8%)	33.3% [11.8%;61.6%]	-
Chickenpox	50 (50.5%)	58 (58.6%)	16.3% [7.32%;29.7%]	-	22 (40.7%)	26 (48.1%)	12.5% [3.51%;29.0%]	-	28 (62.2%)	32 (71.1%)	23.5% [6.81%;49.9%]	-
Tetanus	29 (29.3%)	33 (33.3%)	5.71% [1.58%;14.0%]	-	13 (24.1%)	15 (27.8%)	4.88% [0.60%;16.5%]	-	16 (35.6%)	18 (40.0%)	6.90% [0.85%;22.8%]	-
Influenza	19 (19.2%)	18 (18.2%)	0.89 [0.3;2.6]	1	6 (11.1%)	7 (13.0%)	1.33 [0.23;9.1]	1	13 (28.9%)	11 (24.4%)	0.67 [0.14;2.81]	0,754
Pneumococcal disease	13 (13.1%)	22 (22.2%)	5.5 [1.2;51.07]	0,022	4 (7.41%)	7 (13.0%)	Inf [0.41;Inf]	0,25	9 (20.0%)	15 (33.3%)	4 [0.8;38.67]	0,109
Hepatitis B	59 (59.6%)	63 (63.6%)	10.0% [2.79%;23.7%]	-	30 (55.6%)	33 (61.1%)	12.5% [2.66%;32.4%]	-	29 (64.4%)	30 (66.7%)	6.25% [0.16%;30.2%]	-
						**40 to 60 years old**						
	**2017**	**2022**	**Effectiveness**	**p-value**	**2017**	**2022**	**Effectiveness**	**p-value**	**2017**	**2022**	**Effectiveness**	**p-value**
	N=148	*N=148*			*N=106*	*N=106*			*N=42*	*N=42*		
Measles	94 (63.5%)	106 (71.6%)	22.2% [12.0%;35.6%]	-	64 (60.4%)	75 (70.8%)	26.2% [13.9%;42.0%]	-	30 (71.4%)	31 (73.8%)	8.33% [0.21%;38.5%]	-
Chickenpox	60 (40.5%)	71 (48.0%)	12.5% [6.41%;21.3%]	-	42 (39.6%)	52 (49.1%)	15.6% [7.76%;26.9%]	-	18 (42.9%)	19 (45.2%)	4.17% [0.11%;21.1%]	-

Tetanus	77 (52.0%)	87 (58.8%)	14.1% [6.97%;24.4%]	-	60 (56.6%)	65 (61.3%)	10.9% [3.62%;23.6%]	-	17 (40.5%)	22 (52.4%)	20.0% [6.83%;40.7%]	-
Influenza	36 (24.3%)	55 (37.2%)	3.11 [1.43;7.49]	0,003	17 (16.0%)	34 (32.1%)	5.25 [1.77;21.04]	0,001	19 (45.2%)	21 (50.0%)	1.4 [0.38;5.59]	0,774
Pneumococcal disease	17 (11.5%)	32 (21.6%)	4 [1.46;13.64]	0,004	9 (8.49%)	19 (17.9%)	3.5 [1.1;14.6]	0,031	8 (19.0%)	13 (31.0%)	6 [0.73;275.99]	0,125
Hepatitis B	57 (38.5%)	75 (50.7%)	19.8% [12.2%;29.4%]	-	38 (35.8%)	50 (47.2%)	17.6% [9.47%;28.8%]	-	19 (45.2%)	25 (59.5%)	26.1% [10.2%;48.4%]	-
	**>60 years old**											
	**2017**	**2022**	**Effectiveness**	**p-value**	**2017**	**2022**	**Effectiveness**	**p-value**	**2017**	**2022**	**Effectiveness**	**p-value**
	N=68	*N=68*			*N=54*	*N=54*			*N=14*	*N=14*		
Measles	68 (100%)	68 (100%)	-	-	54 (100%)	54 (100%)	-	-	14 (100%)	14 (100%)	-	-
Chickenpox	11 (16.2%)	19 (27.9%)	14.0% [6.26%;25.8%]	-	7 (13.0%)	13 (24.1%)	12.8% [4.83%;25.7%]	-	4 (28.6%)	6 (42.9%)	20.0% [2.52%;55.6%]	-
Tetanus	47 (69.1%)	55 (80.9%)	38.1% [18.1%;61.6%]	-	37 (68.5%)	45 (83.3%)	47.1% [23.0%;72.2%]	-	10 (71.4%)	10 (71.4%)	0.00% [0.00%;60.2%]	-
Influenza	44 (64.7%)	56 (82.4%)	13 [1.95;552.47]	0,002	34 (63.0%)	45 (83.3%)	Inf [2.51;Inf]	0,001	10 (71.4%)	11 (78.6%)	NA [0;Inf]	1
Pneumococcal disease	11 (16.2%)	18 (26.5%)	4.5 [0.93;42.8]	0,065	6 (11.1%)	11 (20.4%)	3.5 [0.67;34.53]	0,18	5 (35.7%)	7 (50.0%)	2 [0.1;117.99]	0,5
Hepatitis B	13 (19.1%)	23 (33.8%)	18.2% [9.08%;30.9%]	-	7 (13.0%)	15 (27.8%)	17.0% [7.65%;30.8%]	-	6 (42.9%)	8 (57.1%)	25.0% [3.19%;65.1%]	-

Supplementary Table S4. A comparison of the adherence to the recommended vaccination protocol between patients diagnosed with IBD before and after the intervention stratified by age group.

	Total					UC					CD				
	**≤40 years old**														
	**2017**	**2022**	**OR**	**p ratio**	**p overall**	**2017**	**2022**	**OR**	**p ratio**	**p overall**	**2017**	**2022**	**OR**	**p ratio**	**p overall**
	N=99	*N=29*				*N=54*	*N=15*				*N=45*	*N=14*			
Measles	57 (57.6%)	24 (82.8%)	3.43 [1.29;11.1]	0,012	0,024	27 (50.0%)	12 (80.0%)	3.80 [1.05;19.1]	0,042	0,075	30 (66.7%)	12 (85.7%)	2.81 [0.64;21.6]	0,186	0,31
Chickenpox	50 (50.5%)	24 (82.8%)	4.55 [1.71;14.7]	0,002	0,004	22 (40.7%)	12 (80.0%)	5.49 [1.51;27.6]	0,008	0,016	28 (62.2%)	12 (85.7%)	3.40 [0.78;26.0]	0,11	0,188
Tetanus	29 (29.3%)	17 (58.6%)	3.37 [1.43;8.17]	0,005	0,007	13 (24.1%)	8 (53.3%)	3.51 [1.05;12.2]	0,042	0,054	16 (35.6%)	9 (64.3%)	3.16 [0.91;12.2]	0,07	0,112
Influenza	19 (19.2%)	4 (13.8%)	0.69 [0.18;2.08]	0,534	0,696	6 (11.1%)	1 (6.67%)	0.64 [0.02;4.35]	0,687	1	13 (28.9%)	3 (21.4%)	0.69 [0.13;2.74]	0,619	0,738
Pneumococcal disease	13 (13.1%)	6 (20.7%)	1.74 [0.55;4.99]	0,333	0,373	4 (7.41%)	1 (6.67%)	0.98 [0.03;7.80]	0,985	1	9 (20.0%)	5 (35.7%)	2.20 [0.55;8.37]	0,258	0,285
Hepatitis B	59 (59.6%)	21 (72.4%)	1.75 [0.72;4.63]	0,218	0,3	30 (55.6%)	12 (80.0%)	3.05 [0.84;15.3]	0,094	0,156	29 (64.4%)	9 (64.3%)	0.98 [0.28;3.78]	0,98	1
	**40 to 60 years old**														
	**2017**	**2022**	**OR**	**p ratio**	**p overall**	**2017**	**2022**	**OR**	**p ratio**	**p overall**	**2017**	**2022**	**OR**	**p ratio**	**p overall**
	N=148	*N=34*				*N=106*	*N=22*				*N=42*	*N=12*			
Measles	94 (63.5%)	28 (82.4%)	2.62 [1.08;7.47]	0,033	0,057	64 (60.4%)	17 (77.3%)	2.18 [0.78;7.19]	0,141	0,21	30 (71.4%)	11 (91.7%)	3.87 [0.62;103]	0,166	0,254
Chickenpox	60 (40.5%)	22 (64.7%)	2.66 [1.24;5.98]	0,012	0,018	42 (39.6%)	12 (54.5%)	1.82 [0.71;4.72]	0,21	0,293	18 (42.9%)	10 (83.3%)	6.13 [1.37;47.9]	0,016	0,032

Tetanus	77 (52.0%)	15 (44.1%)	0.73 [0.34;1.55]	0,414	0,521	60 (56.6%)	6 (27.3%)	0.29 [0.10;0.78]	0,014	0,023	17 (40.5%)	9 (75.0%)	4.18 [1.04;22.2]	0,043	0,075
Influenza	36 (24.3%)	14 (41.2%)	2.17 [0.98;4.75]	0,057	0,076	17 (16.0%)	8 (36.4%)	2.97 [1.03;8.21]	0,044	0,039	19 (45.2%)	6 (50.0%)	1.21 [0.32;4.58]	0,78	1
Pneumococcal disease	17 (11.5%)	10 (29.4%)	3.20 [1.26;7.82]	0,015	0,017	9 (8.49%)	6 (27.3%)	4.00 [1.17;12.9]	0,028	0,023	8 (19.0%)	4 (33.3%)	2.11 [0.45;8.94]	0,327	0,431
Hepatitis B	57 (38.5%)	21 (61.8%)	2.55 [1.19;5.66]	0,016	0,023	38 (35.8%)	12 (54.5%)	2.13 [0.83;5.56]	0,114	0,163	19 (45.2%)	9 (75.0%)	3.45 [0.87;18.3]	0,081	0,136
						**>60 years old**									
	**2017**	**2022**	**OR**	**p ratio**	**p overall**	**2017**	**2022**	**OR**	**p ratio**	**p overall**	**2017**	**2022**	**OR**	**p ratio**	**p overall**
	N=68	*N=12*				*N=54*	*N=10*				*N=14*	*N=2*			
Measles	68 (100%)	12 (100%)	-	-	-	54 (100%)	10 (100%)	-	-	-	14 (100%)	2 (100%)	-	-	-
Chickenpox	11 (16.2%)	3 (25.0%)	1.76 [0.33;7.25]	0,476	0,432	7 (13.0%)	3 (30.0%)	2.86 [0.49;13.8]	0,223	0,182	4 (28.6%)	0 (0.00%)	-	-	1
Tetanus	47 (69.1%)	9 (75.0%)	1.30 [0.34;6.66]	0,718	1	37 (68.5%)	8 (80.0%)	1.74 [0.37;13.7]	0,508	0,71	10 (71.4%)	1 (50.0%)	0.43 [0.01;19.4]	0,625	1
Influenza	44 (64.7%)	8 (66.7%)	1.07 [0.30;4.53]	0,917	1	34 (63.0%)	6 (60.0%)	0.88 [0.22;3.94]	0,854	1	10 (71.4%)	2 (100%)	-	-	1
Pneumococcal disease	11 (16.2%)	1 (8.33%)	0.53 [0.02;3.25]	0,547	0,681	6 (11.1%)	0 (0.00%)	-	-	0,578	5 (35.7%)	1 (50.0%)	1.73 [0.04;77.4]	0,75	1
Hepatitis B	13 (19.1%)	4 (33.3%)	2.12 [0.48;8.10]	0,3	0,271	7 (13.0%)	2 (20.0%)	1.73 [0.20;9.22]	0,569	0,622	6 (42.9%)	2 (100%)	-	-	0,467
